# Biopsy‐Sparing Diagnosis of Coeliac Disease Based on Endomysial Antibody Testing and Clinical Risk Assessment

**DOI:** 10.1111/apt.70129

**Published:** 2025-04-03

**Authors:** Stiliano Maimaris, Annalisa Schiepatti, Daniel Ignacio Conforme Torres, Roberta Muscia, Virginia Gregorio, Claudia Delogu, Ignazio Marzio Parisi, Michele Dota, Giovanni Arpa, Carolina Cicalini, Giulio Massetti, Chiara Scarcella, Paolo Minerba, Federico Biagi

**Affiliations:** ^1^ Department of Internal Medicine and Medical Therapy University of Pavia Pavia Italy; ^2^ Istituti Clinici Scientifici Maugeri IRCCS, Gastroenterology Unit of Pavia Institute Pavia Italy; ^3^ Department of Molecular Medicine, Unit of Anatomic Pathology University of Pavia Pavia Italy; ^4^ Unit of Anatomic Pathology, ICS Maugeri‐IRCCS SpA SB Pavia Italy

**Keywords:** coeliac disease, duodenal biopsy, endoscopy, gluten, gluten‐free diet, serology, villous atrophy

## Abstract

**Background:**

Interest in a biopsy‐sparing diagnosis of coeliac disease in adults is growing.

**Aims:**

To develop and prospectively validate a non‐invasive diagnostic strategy for adults with suspected coeliac disease based on clinical features and endomysial antibodies (EmA).

**Methods:**

We retrospectively enrolled adults investigated for coeliac disease with EmA and duodenal biopsy between January 2000 and December 2021 in cohort 1 and stratified according to age at presentation (< 45 years; ≥ 45 years) and alarm symptoms. We evaluated diagnostic outcomes and accuracy of EmA. A prospective validation cohort was enrolled between Jan‐2022 and Dec‐2023 (cohort 2).

**Results:**

Cohort 1 included 972 patients (641 F, mean age 42 ± 16); cohort 2 included 214 patients (145 F, 43 ± 18). In cohort 1, 35.4% were diagnosed with coeliac disease and 1.5% with non‐coeliac enteropathies. Of the coeliac disease diagnoses, 173 (50.3%) were in patients < 45 years old without alarm symptoms. No concomitant major organic disorders were diagnosed in patients with coeliac disease. EmA diagnostic accuracy was 99.1% (97.4% sensitivity; 100% specificity and PPV). Regarding non‐coeliac enteropathies, 87% were diagnosed among the 139 patients aged ≥ 45 years old with alarm symptoms and negative EmA. No non‐coeliac enteropathies were diagnosed in patients without alarm symptoms. Findings were confirmed in cohort 2.

**Conclusions:**

Low‐risk adult patients could have been safely diagnosed with coeliac disease non‐invasively based on EmA without endoscopy and duodenal biopsy. Older patients with alarm symptoms should undergo endoscopy with duodenal biopsy to avoid missing non‐coeliac enteropathies. Further validation of our results is necessary.

## Graphical abstract

- We developed and prospectively validated a non‐invasive diagnostic strategy based on age at presentation and alarm symptoms, enabling safe biopsy‐free diagnosis of coeliac disease using endomysial antibodies.
- Younger adults (age < 45 years) without alarm symptoms can be diagnosed non‐invasively based on EmA, while older patients (age ≥ 45 years) with alarm symptoms require endoscopy and duodenal biopsy to avoid missing non‐coeliac enteropathies.

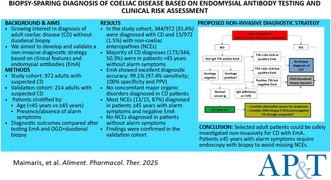

## 1 Introduction

Chronic enteropathies characterised by villous atrophy in adults include coeliac disease and non‐coeliac enteropathies. Coeliac disease is a chronic immune‐mediated enteropathy triggered by gluten in genetically predisposed individuals [1, 2, 3, 4] which has a prevalence of approximately 1% in the general population of western countries [5]. It is characterised by specific circulating antibodies and is clinically heterogeneous, ranging from a severe malabsorption syndrome to mild extra‐intestinal symptoms, or even asymptomatic cases detected by screening [1, 2, 3, 4]. Non‐coeliac enteropathies are rare disorders of various aetiologies including autoimmune enteropathy, common‐variable immune deficiency, infectious and iatrogenic causes and are characterised clinically by severe malabsorption due to villous atrophy [6]. They are unrelated to gluten consumption and lack coeliac antibodies, posing a difficult differential diagnosis with seronegative coeliac disease, a rare form of coeliac disease [1, 2, 3, 4, 6].

In adults, current guidelines recommend that coeliac disease should be diagnosed in adults based on positive IgA tissue transglutaminase antibodies (TTA)/endomysial antibodies (EmA) and a certain degree of villous atrophy on duodenal biopsies while the patient is on a gluten‐containing diet [1, 2, 3, 4]. On the contrary, in a paediatric setting, a no‐biopsy diagnostic approach has been adopted for selected patients since 2012 based on TTA levels > 10× the upper limit of normality [7, 8]. There has recently been great interest in potentially implementing a no‐biopsy strategy also in adults and many studies, mainly focusing on TTA, have shown promising results [9, 10, 11, 12, 13, 14, 15, 16, 17, 18, 19, 20, 21, 22, 23, 24, 25, 26]. Moreover, a recent meta‐analysis found that TTA > 10× upper limit of normal have a specificity of 100% and a PPV of 98% in an adult secondary care setting [27], reaching very similar specificity to EmA [28].

However, this has not yet led to widespread adoption of a no‐biopsy strategy in adults [1, 2, 3, 4], barring a few exceptions, such as the Finnish national guidelines [29], which already allow for a no‐biopsy strategy in adults, and the 2020 interim guidance by the British Society of Gastroenterology, which allowed a no‐biopsy diagnosis in selected cases during the COVID‐19 pandemic [30].

Nevertheless, biopsy‐sparing diagnosis of coeliac disease in adults has some issues. Firstly, seronegative coeliac disease and non‐coeliac enteropathies cannot be diagnosed without a biopsy, and differential diagnosis can be very challenging. Moreover, there are concerns about missing concomitant organic pathologies in coeliac patients or synchronous malignant complications of coeliac disease, although this has been shown to be extremely rare [31, 32], with these complications developing mainly in patients diagnosed in older adulthood (≥ 45 years) [33, 34]. Finally, symptoms of coeliac disease in adult patients overlap significantly with those of many other gastrointestinal conditions, including malignancies and many patients with suspected coeliac disease require upper endoscopy not only for duodenal biopsy to confirm/exclude coeliac disease but also to rule out other conditions unrelated to coeliac disease.

Over the last 20 years at our centre, we have followed a standard diagnostic approach for patients with a suspicion of coeliac disease based on IgA EmA testing at our laboratory and duodenal biopsy while on a gluten‐containing diet. We aimed to retrospectively develop and prospectively validate on a separate cohort a non‐invasive diagnostic strategy for coeliac disease based on patient clinical features and EmA to guide clinicians in identifying patients at low risk for non‐coeliac enteropathies/comorbid pathology who can be safely studied non‐invasively for suspected coeliac disease versus high‐risk patients in need of upper GI endoscopy and duodenal biopsy. Secondarily, we also aimed to estimate the fraction of patients with a suspicion of coeliac disease or other chronic enteropathies who could safely avoid undergoing duodenal biopsy.

## 2 Patients and Methods

### 2.1 Study Design, Setting and Study Population

This is a single‐centre observational study consisting of two cohorts. Cohort 1 (the study cohort) consists of consecutive adult patients (age ≥ 18 years) tested with both IgA EmA and upper endoscopy with duodenal biopsy at our centre while on a gluten containing diet (GCD) for a suspicion of chronic enteropathies between January 2000 and December 2021 and retrospectively enrolled. Any patients who did not undergo testing with IgA EmA at our laboratory and duodenal biopsy at our endoscopy unit were excluded from cohort 1.

Cohort 2 (the validation cohort) consisted of consecutive patients prospectively evaluated at our centre in whom there was suspicion of coeliac disease or other chronic enteropathies between January 2022 and December 2023. Patients who were tested serologically for coeliac disease and/or referred for duodenal biopsy were included.

To avoid potential biases, we excluded from both cohorts all patients referred from other centres for confirmation or re‐evaluation of a previous diagnosis of coeliac disease/non‐coeliac enteropathies, for suspected refractory/complicated coeliac disease, and any patients who had already started a GFD and needed to be re‐evaluated after gluten challenge.

### 2.2 Diagnostic Testing for Coeliac Disease

IgA EmA were detected on monkey oesophagus/jejunum sections using an indirect immunofluorescence kit (INOVA diagnostics, San Diego, CA). An EmA titre ≥ 1:5 was considered positive. We have previously shown EmA to have excellent diagnostic accuracy in our hands, which is substantially similar to TTA [35]. Total serum immunoglobulins were tested to exclude immunoglobulin deficiencies, which may give false negative results. During upper endoscopy, at least four biopsies were taken from the second portion of the duodenum, oriented on nitrate‐cellulose paper and subsequently formalin‐fixed, paraffin embedded, and cut perpendicularly to the luminal surface for histological evaluation.

### 2.3 Diagnostic Criteria for Coeliac Disease and Non‐Coeliac Enteropathies

We specify that over the last 20 years at our centre we have been adopting the policy of routinely performing IgA EmA in all patients with suspicion of chronic enteropathies on a serum sample taken the same day as diagnostic upper GI endoscopy with duodenal biopsy. Moreover, duodenal specimens are carefully oriented in order to avoid pitfalls due to incorrect processing of duodenal biopsies.

Diagnosis of conventional seropositive coeliac disease was made based on a certain degree of villous atrophy on correctly‐oriented biopsies from the second portion of the duodenum and positive IgA EmA [1, 2, 3, 4]. Diagnosis of seronegative coeliac disease was made after exclusion of all other possible causes of villous atrophy with negative coeliac serology and confirmed by histological recovery of villous atrophy on a GFD [6, 36]. Serum IgA levels were tested in all patients and, in case of total IgA deficiency, IgG EmA were tested. If positive, this was considered supportive of a diagnosis of coeliac disease. HLA DQ2/DQ8 further supported the diagnosis of seronegative coeliac disease and coeliac disease + IgA deficiency [6, 36]. Potential coeliac disease was diagnosed in patients with positive EmA but no evidence of villous atrophy on duodenal biopsies [1, 2, 3, 4].

Diagnosis of non‐coeliac enteropathies was made in accordance with specific criteria for each disorder. Diagnosis of autoimmune enteropathy was based on persistent severe villous atrophy and malabsorption not responsive to a GFD and positive enterocyte antibodies [6, 36]. Common variable immune deficiency was diagnosed in accordance with international diagnostic criteria [6, 36]. Iatrogenic enteropathies were diagnosed in patients with a suggestive pharmacological history, observation of clinical/histological response following drug withdrawal, and after exclusion of other causes of villous atrophy [6]. Infectious enteropathy was diagnosed in patients with villous atrophy due to an infectious agent [6, 36]. Diagnosis of small bowel lymphomas and small bowel carcinomas was based on a combination of imaging tests (abdominal CT/MRI/PET) and histopathology [6]. Diagnosis of Whipple's disease was made based on a finding of PAS‐positive diastase resistant foamy macrophage infiltration of the duodenal lamina propria [37]. Patients with persistent seronegative villous atrophy in whom all other causes of villous atrophy were excluded after extensive investigations were considered to be affected by a form of persistent idiopathic villous atrophy [6]. Finally, patients with normal duodenal villous architecture and negative EmA were considered not to be affected by enteropathy.

### 2.4 Clinical Risk Stratification for Need of Invasive Evaluation

We evaluated diagnostic outcomes according to EmA results, age at presentation (< 45 vs. ≥ 45 years) and presence of alarm symptoms, which are major risk factors for organic gastrointestinal disorders and complications of coeliac disease [33]. The age threshold of 45 years was selected based on previous research indicating a substantially increased risk of complications in coeliac disease patients diagnosed after this age [34]. For the purposes of this study, we considered alarm symptoms to be the following: weight loss, anaemia of any type, dysphagia, recurrent vomiting, symptoms awaking patients at night, fever and a family history of gastrointestinal cancer. Final diagnoses reached were then compared based on EmA results, age at presentation and presence of alarm symptoms.

### 2.5 Data Collection

For all enrolled patients the following data were retrospectively collected from clinical notes: age at enrolment (diagnostic duodenal biopsy), sex, presenting symptoms (including alarm symptoms), medical and family history, laboratory test results including EmA, TTA and serum immunoglobulins, upper GI endoscopy findings and duodenal histology, and, if available, HLA‐DQ typing.

### 2.6 Ethics

The study was conducted in accordance with the Declaration of Helsinki (6th revision, 2008). All diagnostic procedures were performed as part of routine clinical practice. The study protocol was approved by the Ethics Committee of IRCCS Pavia, ICS Maugeri, Pavia, Italy (protocol number 2381 ce, approved on 14 January 2020).

### 2.7 Statistical Analysis

Statistical analysis was performed using R version 4.3.1 (R Core Team (2022). R: A language and environment for statistical computing. R Foundation for Statistical Computing, Vienna, Austria. URL https://www.R‐project.org/). Categorical variables were summarised as total counts and percentages and compared among groups with Fisher's exact test. Continuous variables were summarised as mean and standard deviation (SD) or median and interquartile range (IQR), as appropriate. Continuous variables were compared among groups using the unequal variances t‐test, the Mann–Whitney *U* test, the Kruskal–Wallis test or one‐way ANOVA, as appropriate. Post hoc pairwise comparisons were adjusted for multiplicity using the Benjamini‐Hochberg false discovery method. The diagnostic accuracy, sensitivity, specificity, positive predictive value and negative predictive value of EmA were calculated with 95% confidence intervals (95% CI). Two‐sided *p*‐values < 0.05 were considered statistically significant for all analyses.

## 3 Results

**FIGURE 1:**
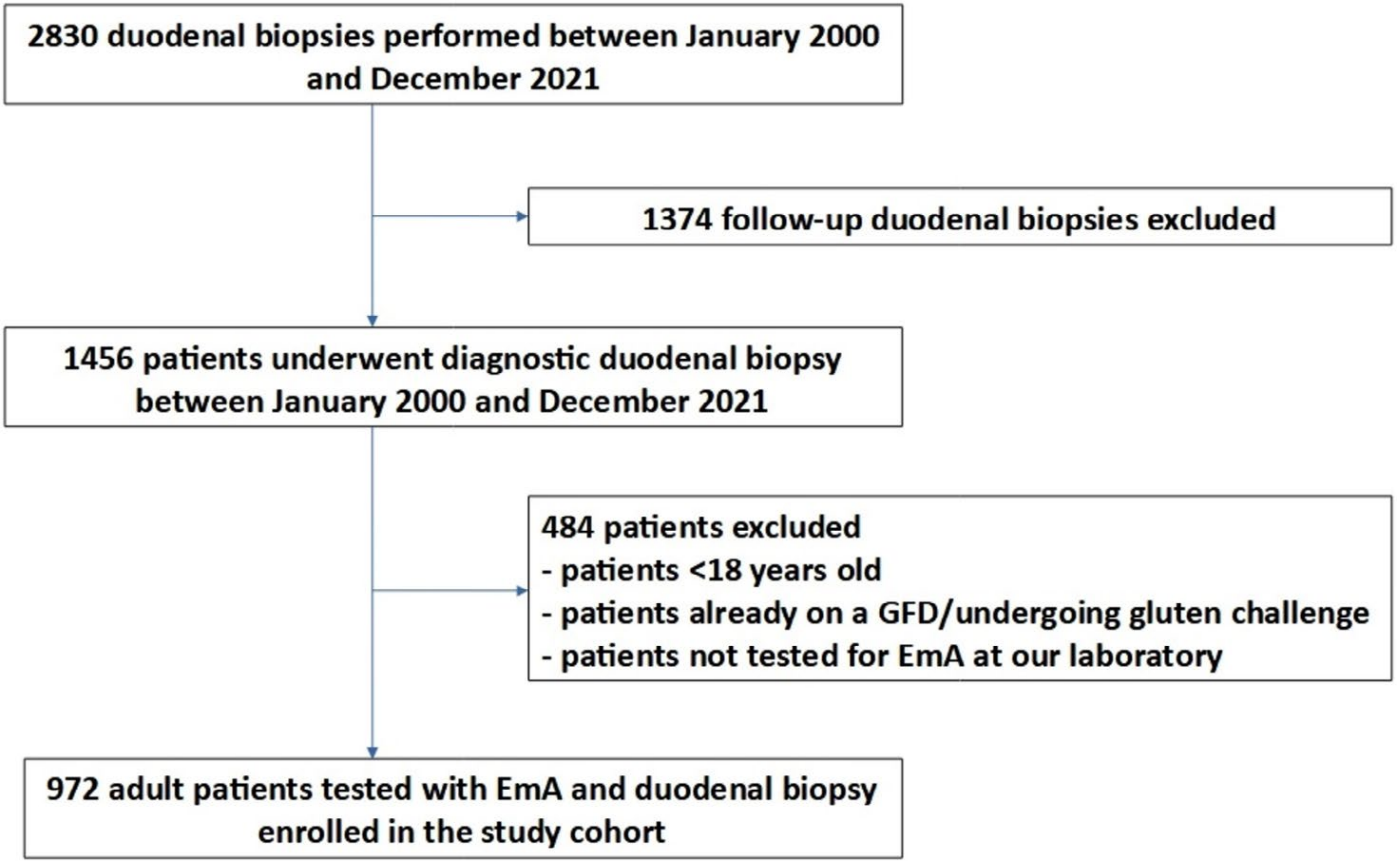
Flowchart showing patients included and excluded from the study cohort and reasons for exclusion. EmA, endomysial antibodies; GFD, gluten‐free diet.

The flowchart in Figure 1 shows patients included and excluded from cohort 1 (the study cohort) and reasons for exclusion. Overall, 972 patients (641 F, mean age 42 ± 16 years) were included in cohort 1. Baseline clinical and demographic features of patients in cohort 1 are shown in Table 1. The majority of patients were investigated due to gastrointestinal symptoms such as diarrhoea (41.9%), weight loss (26.0%), abdominal pain (29.7%) and dyspepsia (27.8%) or other signs of malabsorption such as anaemia (19.3%). A minority had a family history of coeliac disease (13.4%) or had associated autoimmune conditions (7.5%).

**TABLE 1:** Clinical characteristics of patients overall and comparison according to final diagnosis.

	All patients *N* = 972	Coeliac disease^a^ *N* = 344	Non‐coeliac enteropathies^b^ *N* = 15	No enteropathy *N* = 613	*p*
Age, years (mean ± SD)	42 ± 16	36 ± 13	59 ± 14	45 ± 16	< 0.001
Sex, F (%)	641 (65.8%)	231 (67.2%)	6 (40.0%)	404 (65.9%)	0.10
Diarrhoea (%)	379 (41.6%)	102 (30.9%)	11 (73.3%)	268 (47.4%)	< 0.001
Weight loss (%)	229 (25.1%)	56 (17.0%)	13 (86.7%)	167 (29.6%)	< 0.001
Anaemia (%)	171 (18.8%)	81 (24.5%)	6 (40.0%)	89 (15.8%)	< 0.01
Abdominal pain (%)	266 (29.2%)	69 (20.9%)	4 (26.7%)	197 (34.9%)	< 0.001
Dyspepsia (%)	248 (27.2%)	50 (15.2%)	5 (33.3%)	198 (35.0%)	< 0.001
Dermatitis herpetiformis (%)	31 (3.4%)	31 (9.4%)	0 (0.0%)	0 (0.0%)	< 0.001
Autoimmune conditions (%)	68 (7.5%)	28 (8.5%)	1 (6.7%)	39 (6.9%)	0.63
First degree family history of coeliac disease (%)	123 (13.5%)	70 (21.1%)	0 (0.0%)	52 (9.2%)	< 0.001
Immunosuppressive drug treatment (%)	20 (2.1%)	5 (1.4%)	2 (11.8%)	13 (2.1%)	0.048
Hospitalisation (%)	83 (8.5%)	10 (2.9%)	9 (52.9%)	64 (10.4%)	< 0.001
Positive EmA (%)	335 (34.4%)	335 (97.4%)	0 (0.0%)	0 (0.0%)	< 0.001
Immunoglobulin deficiency (%)	5 (0.5%)	3 (0.9%)	2 (13.3%)	0 (0.0%)	0.04
Villous atrophy at duodenal biopsy (%)	316 (32.4%)	302 (87.8%)	13 (86.7%)	0 (0.0%)	< 0.001

Abbreviations: EmA, endomysial antibodies; F, female; SD, standard deviation.

^a^ Includes 296 patients with conventional seropositive coeliac disease, 42 with potential coeliac disease, 3 with coeliac disease + total IgA deficiency and 3 with seronegative coeliac disease.

^b^ Includes 6 patients with drug‐related enteropathies (5 due to angiotensin receptor blockers, 1 due to immune checkpoint inhibitors), 4 patients with intestinal lymphomas unrelated to coeliac disease (3 T‐cell, 1 B‐cell), 1 patient with common variable immune deficiency, 2 patients with Whipple's disease, 1 with infectious enteropathy, and 3 with idiopathic villous atrophy.

After all investigations, different forms of coeliac disease were diagnosed in 344 patients (35.4%), non‐coeliac enteropathies were diagnosed in 15 patients (1.7%) and enteropathy was excluded in the remaining 613 patients (62.9%). More precisely, among the 344 coeliac patients, 296 had conventional seropositive coeliac disease, 42 had potential coeliac disease, 3 had coeliac disease + total IgA deficiency and 3 had seronegative coeliac disease. Conversely, the 15 patients with non‐coeliac enteropathies included 6 patients with drug‐related enteropathies (5 due to ARBs, 1 due to immune checkpoint inhibitors), 3 patients with intestinal lymphomas unrelated to coeliac disease (2 T‐cell, 1 B‐cell), 1 patient with common variable immune deficiency, 1 patient with Whipple's disease, 1 with infectious enteropathy and 3 with idiopathic villous atrophy.

### 3.1 Endomysial Antibodies Testing

Table 2 summarises data on the diagnostic performance of EmA for diagnosis of coeliac disease in cohort 1. Overall, in cohort 1, 335 patients had positive IgA EmA and 637 had negative IgA EmA. All the 335 patients with positive EmA had either conventional seropositive coeliac disease (293 patients) or potential coeliac disease (42 patients). Among the 637 patients with negative IgA EmA, 9 had coeliac disease, of which 3 had total IgA deficiency, 3 had seronegative coeliac disease and 3 had false negative EmA results (TTA were positive). Overall, the diagnostic accuracy of EmA for coeliac disease was 99.1% (95% CI 98.2%–99.6%), the sensitivity of EmA was 97.4% (95% CI 95.1%–98.8%) and the specificity was 100% (95% CI 99.1%–100%). The positive predictive value of EmA for coeliac disease was 100% (95% CI 98.4%–100%) while the negative predictive value of EmA for coeliac disease was 98.6% (95% CI 97.3%–99.4%).

**TABLE 2:** Diagnostic performance of endomysial antibodies for the diagnosis of coeliac disease in the study cohort (cohort 1).

	Study cohort (cohort 1) total *N* = 972
True positives (%)	335^a^ (34.4%)
False positives (%)	0 (0%)
True negatives (%)	628 (64.6%)
False negatives (%)	9 (0.9%)^b^
Overall accuracy (95% CI)	99.1% (98.2%–99.6%)
Sensitivity (95% CI)	97.4% (95.1%–98.8%)
Specificity (95% CI)	100% (99.1%–100%)
Positive predictive value (95% CI)	100% (98.4%–100%)
Negative predictive value (95% CI)	98.6% (97.3%–99.4%)

^a^ Includes 42 patients diagnosed with potential coeliac disease.

^b^ Three patients had total IgA deficiency, Three had seronegative coeliac disease and Three had false negative endomysial antibody results (tissue transglutaminase antibodies were positive).

### 3.2 Diagnostic Outcomes According to Clinical Features and Endomysial Antibodies Results

Table 3 shows a comparison of overall clinical features and diagnostic outcomes according to patient age at presentation (< 45 years vs. ≥ 45 years) and presence of alarm symptoms. Overall, all patients with positive EmA were diagnosed with coeliac disease and none of the patients diagnosed with coeliac disease had any concomitant major organic gastrointestinal disorders or complications of coeliac disease at the time of diagnosis, regardless of age at presentation or presence of alarm symptoms.

**TABLE 3:** Comparison of the clinical features and final diagnoses reached according to age at presentation and presence of alarm symptoms in cohort 1.

	Age < 45 years (*N* = 588)		Age ≥ 45 years (*N* = 384)		*p*
	Without alarm symptoms (*N* = 359)	With alarm symptoms (*N* = 229)	Without alarm symptoms (*N* = 213)	With alarm symptoms (*N* = 171)	
Age, years (mean ± SD)	32 ± 8	32 ± 8	58 ± 10	60 ± 11	< 0.001
Sex, F (%)	228 (63.5%)	180 (78.6%)	126 (59.2%)	107 (62.6%)	< 0.001
Diarrhoea (%)	126 (39.1%)	91 (39.7%)	88 (46.8%)	76 (44.4%)	0.288
Weight loss (%)	0 (0.0%)	123 (53.7%)	0 (0.0%)	114 (66.7%)	< 0.001
Anaemia (%)	0 (0.0%)	106 (46.3%)	0 (0.0%)	70 (40.9%)	< 0.001
Abdominal pain (%)	99 (30.7%)	69 (30.1%)	54 (28.7%)	48 (28.1%)	0.926
Dyspepsia (%)	86 (26.7%)	53 (23.1%)	62 (33.0%)	52 (30.4%)	0.123
Dermatitis herpetiformis (%)	21 (6.5%)	4 (1.7%)	5 (2.7%)	1 (0.6%)	< 0.01
Autoimmune conditions (%)	28 (8.7%)	10 (4.4%)	18 (9.6%)	12 (7.0%)	0.14
1st degree family history of coeliac disease (%)	63 (19.6%)	17 (7.4%)	29 (15.4%)	13 (7.6%)	< 0.001
Dysphagia (%)	0 (0.0%)	3 (1.3%)	0 (0.0%)	3 (1.8%)	0.02
Recurrent vomiting (%)	0 (0.0%)	24 (10.5%)	0 (0.0%)	11 (6.4%)	< 0.001
Fever (%)	0 (0.0%)	3 (1.3%)	0 (0.0%)	5 (2.9%)	< 0.01
Family history of gastrointestinal cancer (%)	0 (0.0%)	3 (1.3%)	0 (0.0%)	6 (3.5%)	0.001
Immunosuppressive drug treatment (%)	5 (1.4%)	1 (0.4%)	5 (2.3%)	9 (5.3%)	< 0.01
Immunoglobulin deficiency (%)	1 (0.3%)	1 (0.4%)	1 (0.5%)	1 (0.6%)	0.92
Positive EmA (%)	168 (46.8%)	93 (40.6%)	42 (19.7%)	32 (18.7%)	< 0.001
Villous atrophy at duodenal biopsy (%)	151 (42.1%)	89 (38.9%)	36 (16.9%)	39 (22.8%)	< 0.001
Final diagnosis (%)					
Coeliac disease	173 (48.2%)	93 (40.6%)	44 (20.7%)	34 (19.9%)	< 0.001
No enteropathy	186 (51.8%)	134 (58.5%)	169 (79.3%)	124 (72.5%)	
NCE	0 (0.0%)	2 (0.9%)	0 (0.0%)	13 (7.6%)	

Abbreviations: EmA, endomysial antibodies; F, female; NCE, non‐coeliac enteropathies; SD, standard deviation.

**FIGURE 2:**
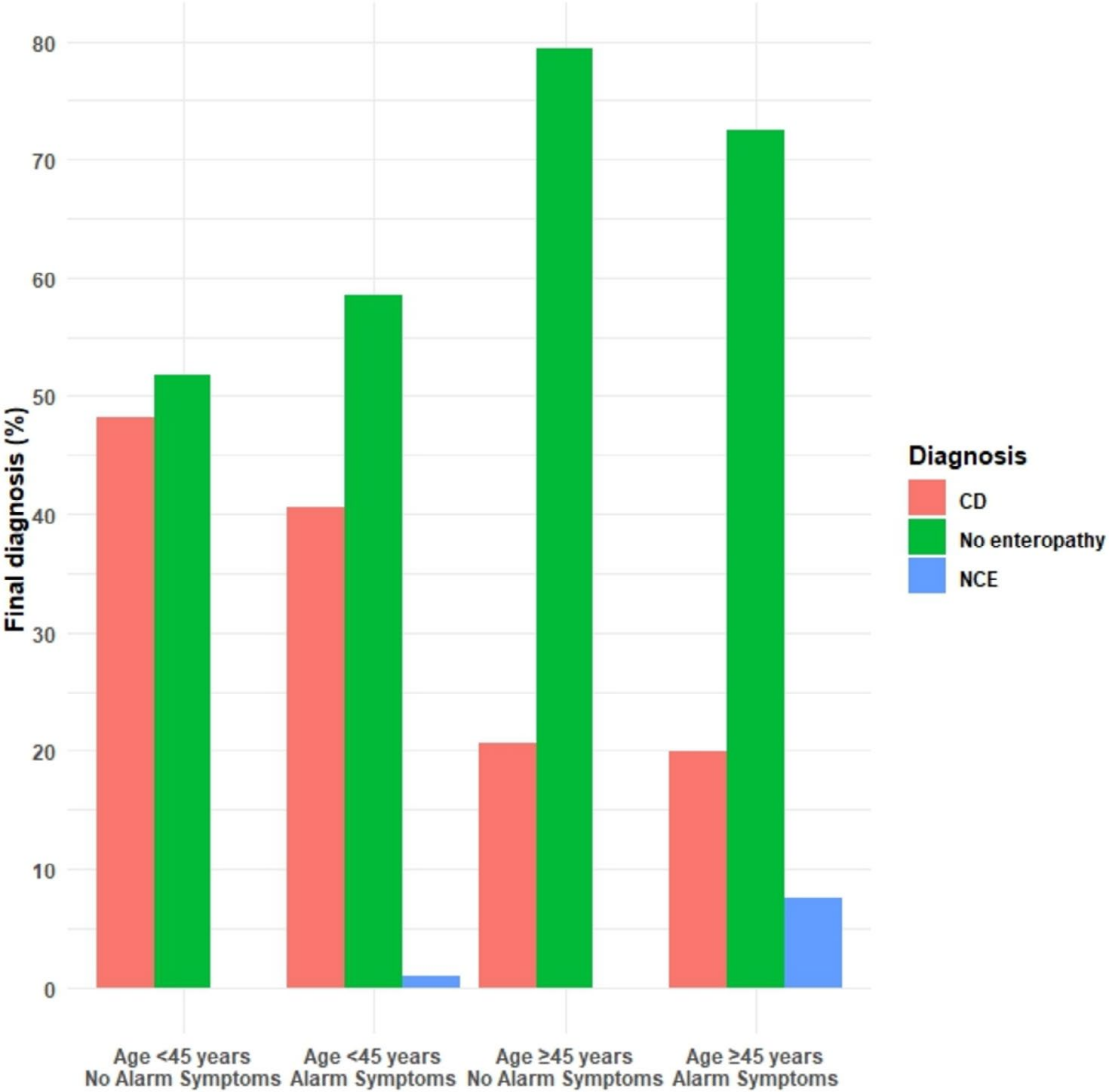
Comparison of diagnostic outcomes in patients investigated for suspected enteropathy with endomysial antibodies and upper endoscopy with duodenal biopsies according to age at presentation and presence of alarm symptoms. CD, coeliac disease; NCE, non‐coeliac enteropathy.

Secondly, as shown in Figure 2, most coeliac disease diagnoses (173/344, 50.2%) were made in patients < 45 years without alarm symptoms. Moreover, only a minority of patients diagnosed with coeliac disease were ≥ 45 years old at presentation (78/344, 22.7%). On the contrary, the large majority of non‐coeliac enteropathies (13/15, 86.7%) were diagnosed among patients ≥ 45 years old with alarm symptoms at presentation. None of the patients diagnosed with non‐coeliac enteropathies had positive EmA. When considering only the 139 patients ≥ 45 years old with alarm symptoms and negative EmA, the prevalence of non‐coeliac enteropathies was 13/139 (9.4%). No patients were diagnosed with non‐coeliac enteropathies among those under 45 years of age without alarm symptoms. Finally, of the three patients diagnosed with seronegative coeliac disease, one was < 45 years old at diagnosis and was diagnosed due to dermatitis herpetiformis, while the other two patients were ≥ 45 years old (one with alarm symptoms, the other without).

### 3.3 Need for Invasive Evaluation According to Clinical Characteristics and Serological Results

EmA positive patients all had coeliac disease and none had concomitant major gastrointestinal organic disorders or complications of coeliac disease. This remained the case regardless of age at presentation or presence of alarm symptoms, suggesting that invasive evaluation for confirmation of coeliac disease diagnosis or exclusion of concomitant pathology in patients with positive IgA EmA is of limited value. On the contrary, we found a high prevalence of non‐coeliac enteropathies (13/139, 9.4%) in patients presenting at age ≥ 45 years with alarm symptoms and negative EmA. This also included malignant intestinal lymphomas, so upper GI endoscopy with duodenal biopsy in this group of patients is absolutely necessary to rule out non‐coeliac enteropathies and malignancies.

Finally, it is noteworthy that two patients with non‐coeliac enteropathies had low‐titre positive TTA but negative EmA. One of them had olmesartan enteropathy with excellent clinical and histological response to olmesartan withdrawal, while the other was diagnosed with idiopathic villous atrophy after showing no clinical/histological response to a GFD and exclusion of other causes. Therefore, patients with low‐titre positive TTA but negative EmA should be carefully investigated and require upper GI endoscopy and duodenal biopsy to avoid diagnostic errors.

### 3.4 Prospective Validation on Cohort 2

Overall, 214 patients (145 F, mean age 43 ± 18) were included in cohort 2. The main reasons for suspecting coeliac disease in these patients included diarrhoea (61.2%), weight loss (21.5%), anaemia (13.1%), associated autoimmune conditions (15.0%) and a family history of coeliac disease (8.9%). In 160/214 (74.8%) screening for coeliac disease with IgA TTA was performed and was negative, and no further investigations for enteropathy were performed in these patients. The remaining 54 patients (25.2%) were further investigated for enteropathy and underwent upper GI endoscopy with duodenal biopsy and IgA EmA testing at our laboratory. Reasons for further investigation beyond TTA screening in these 54 patients were positive TTA (21 patients), strong clinical suspicion for enteropathy due to the clinical picture (32 patients) and the presence of immunoglobulin deficiency (1 patient).

Of these 54 patients, 21 were diagnosed with coeliac disease (including one patient diagnosed with potential coeliac disease), while in the remaining 33 patients, enteropathy was excluded. None were diagnosed with non‐coeliac enteropathies. Regarding the 33 patients without enteropathy, colonoscopy revealed inflammatory bowel disease in 2 of them and colitis due to pembrolizumab in 1 patient, while in the remaining 30 an organic cause was excluded. Of the 21 patients with coeliac disease, the majority (13/21, 61.9%) were < 45 years old at presentation. More in detail, 5/21 were < 45 years old without alarm symptoms, 8/21 were < 45 years with alarm symptoms, 3 were ≥ 45 years old without alarm symptoms, and finally 5/21 were ≥ 45 years old with alarm symptoms.

All patients diagnosed with coeliac disease had positive IgA EmA and none of the patients in whom enteropathy was excluded had positive EmA. The overall diagnostic accuracy of EmA for coeliac disease in the validation cohort was 100% (95% CI 90.3%–100%), sensitivity was 100% (95% CI 77.2%–100%) and specificity was 100% (95% CI 84.7%–100%). The positive predictive value was 100% and negative predictive value was also 100%.

## 4 Discussion

This large single‐centre real‐world study provides important insights to guide clinical decision‐making and an initial proposal towards future non‐invasive diagnosis of coeliac disease in adults. Our findings support the feasibility of a biopsy‐sparing approach for diagnosing coeliac disease in low‐risk adults with positive EmA, while also emphasising the importance of endoscopic evaluation in older patients with alarm symptoms to rule out non‐coeliac enteropathies and malignancies. This could allow avoidance of invasive and costly procedures in a significant portion of patients.

We propose an accurate non‐invasive diagnostic strategy based on age at presentation, presence of alarm symptoms, and EmA in order to identify coeliac patients and discriminate them from those affected by non‐coeliac enteropathies and those without enteropathy. The majority of coeliac patients (50.2%) were < 45 years old without alarm symptoms, and all patients with positive EmA were diagnosed with coeliac disease. On the contrary, the large majority (86.7%) of non‐coeliac enteropathies were diagnosed in patients ≥ 45 years old with alarm symptoms. Moreover, no cases of non‐coeliac enteropathies or malignancies were diagnosed in patients without alarm symptoms. The high prevalence of these conditions in older patients with alarm symptoms underscores the continued need for endoscopic evaluation in high‐risk individuals with negative EmA. Our results are also in accordance with those of a recent large UK‐based study on 382,370 patients undergoing upper GI endoscopy, which found that in patients under 50 years of age the overall cancer rate was extremely low (< 0.1%) [38]. Moreover, in those under 50, cancer rates were always < 1% even in patients with alarm symptoms, strongly suggesting the possibility to avoid upper GI endoscopy in younger patients, even when presenting with alarm symptoms [38]. This is particularly important considering that in our study the large majority of coeliac patients were diagnosed at age < 45 (266/344, 77.3%), with most also being < 45 years old without any alarm symptoms (173/344, 50.2%).

The present study is the first to investigate the role of age and alarm symptoms in adult patients with suspected coeliac disease in consideration of a potential biopsy‐sparing approach. Apart from the British Society of Gastroenterology interim guidance during the COVID‐19 pandemic, which set the age cut‐off of < 50 years for potentially avoiding duodenal biopsy [20], there is currently no other real‐world data suggesting what the best age cut‐off may be. The available literature shows that age is a major clinical risk factor for negative outcomes, particularly persistence of villous atrophy and risk of refractory coeliac disease and other malignant complications [33, 34, 39]. However, all these papers were studies investigating the follow‐up of coeliac disease, and not the diagnosis of coeliac disease, and all had as a primary endpoint the evaluation of the risk of complications and mortality.

Seronegative coeliac disease was rare in our study and although it is important to consider seronegative coeliac disease in high‐risk patients with negative serology, this suggests that the risk of missing seronegative coeliac disease is very low in young adults without alarm symptoms. Frequently, there are also other clues pointing towards the possibility of seronegative coeliac disease or non‐coeliac enteropathies that should be noted, such as immunoglobulin deficiency or a suggestive pharmacological history (e.g., olmesartan use) [6, 40].

As it has been long‐established, EmA have excellent diagnostic accuracy and high specificity [28]. This was confirmed in our study with EmA showing virtually perfect specificity (100%) and positive predictive value (100%) for coeliac disease diagnosis in both the study and validation cohorts, supporting the reliability of EmA‐based diagnosis without biopsy confirmation in appropriately selected adult patients. Our results suggest that young adults (age < 45 years) without alarm symptoms could be safely evaluated non‐invasively for coeliac disease. Applying such a strategy would have led to no missed diagnoses in either the study cohort nor the validation cohort. Moreover, none of the patients diagnosed with coeliac disease had concomitant major organic gastrointestinal disorders or complications of coeliac disease at the time of diagnosis, so the value of performing upper endoscopy with duodenal biopsy in confirming coeliac disease in patients with positive EmA appears limited. Considering that our study included more than 1000 patients investigated over more than two decades, this strongly suggests that a biopsy‐sparing strategy for coeliac disease can be safely and reliably applied in a substantial proportion of patients. On the contrary, the prevalence of non‐coeliac enteropathies was high (almost 10%) in patients ≥ 45 years old with alarm symptoms and negative EmA. Severe malabsorption symptoms or other suggestive clinical clues for seronegative enteropathies (e.g., olmesartan use, immunoglobulin deficiency) are often present and should prompt endoscopy with duodenal biopsies.

**FIGURE 3:**
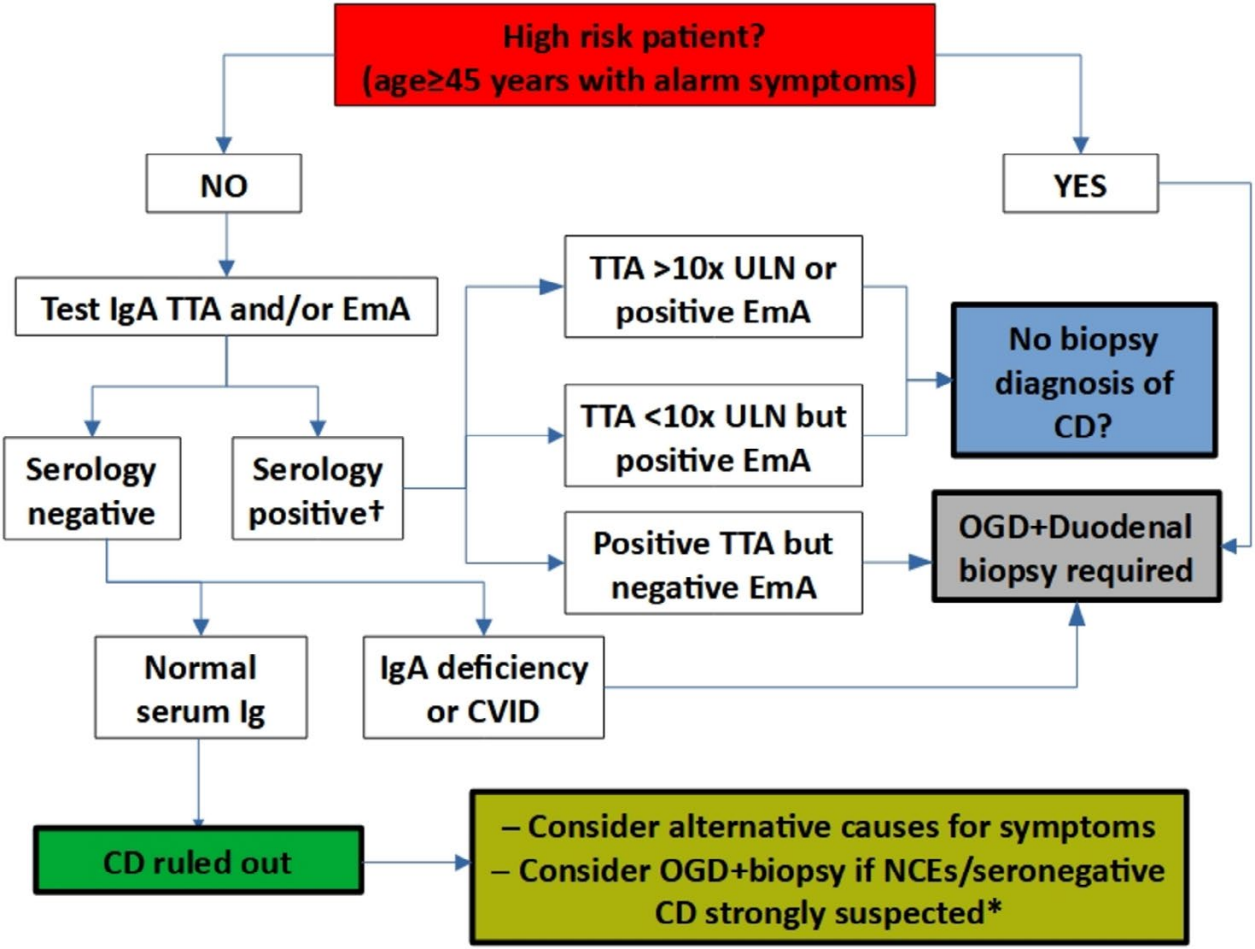
Proposed diagnostic strategy for future research on non‐invasive evaluation of adult patients with suspected coeliac disease incorporating EmA based on our results and the available literature data. CD, coeliac disease; CVID, common variable immune deficiency; EmA, endomysial antibodies; IgA, immunoglobulin A; OGD, oesophagogastroduodenoscopy; TTA, tissue transglutaminase antibodies; ULN, upper limit of normality. *If non‐coeliac enteropathies or seronegative coeliac disease are strongly suspected due to severe malabsorption or other clinical clues, consider upper endoscopy with duodenal biopsy. Otherwise, consider testing for alternative causes of symptoms besides enteropathy in this group of patients. †For centres lacking access to EmA, we propose diagnosing coeliac disease without duodenal biopsy only in patients with positive TTA > 10× upper limit of normal and performing duodenal biopsy in patients with positive TTA < 10× upper limit of normal.

In Figure 3, based on our results and data from the literature, we propose a potential diagnostic strategy for future research on non‐invasive evaluation of patients with suspected coeliac disease that incorporates EmA. Ultimately, we believe EmA can play an important role in the non‐invasive investigation of patients with suspected coeliac disease, including also those with low titre TTA, below the threshold of 10x upper limit of normal, which actually represent the majority of coeliac patients [27]. This approach could substantially reduce the need for invasive procedures in patients with suspected coeliac disease. Given that data from meta‐analyses in the literature report levels of specificity > 99% for both TTA > 10× upper limit of normal [27] and EmA [28], we believe that their diagnostic value is virtually identical, with both essentially guaranteeing that a diagnosis of coeliac disease will be confirmed. However, future multicentre prospective validation of our results is necessary to ensure the generalisability of our results, since our data is based on results from a single tertiary centre specialised in the diagnosis of coeliac disease and non‐coeliac enteropathies. It is also important to emphasise that although in our study EmA showed excellent diagnostic accuracy and that clear diagnostic patterns emerged based on age at presentation, alarm symptoms, and EmA results, further research in more diverse clinical settings is necessary to confirm our findings before they can be extrapolated into everyday clinical practice.

Another interesting point regards potential coeliac disease, which we found affected approximately 10% of EmA‐positive patients in the study cohort, but only one patient in the validation cohort. For the purposes of the study, we considered these patients as being affected by a form of coeliac disease rather than as not affected by enteropathy. This is also supported by findings on adult patients with potential coeliac disease [41] and the results of a recent meta‐analysis on outcomes in patients diagnosed with potential coeliac disease [42]. Although major international guidelines [1, 2, 3, 4] recommend taking bulb biopsies as part of the diagnostic workflow for coeliac disease, unfortunately, adherence to these recommendations is still low worldwide, as shown by a recently published international multicentre study on ultrashort coeliac disease [43]. In this regard, our centre, which actively took part in the multicentre study by Raju et al. is no exception, as we have been adopting the policy to perform bulb biopsy based on endoscopic appearance of the duodenal bulb. Therefore, we cannot say how many patients affected by potential coeliac disease may actually have been diagnosed with ultrashort coeliac disease if bulb biopsies had been taken systematically. Based on previously published data, the estimated prevalence of ultrashort coeliac disease in our centre is roughly 0.8% in our centre [43].

Although our study has several strengths, including the large sample size spanning a long period of time and a prospective validation cohort, there are also several limitations we must acknowledge. These are mainly related to the single‐centre study design and the use of EmA, which may limit generalisability of our results to centres where EmA is not available. Although our results with EmA were excellent, the use of EmA is also the most relevant limitation, potentially impacting on the reproducibility of our results on a larger scale, both because EmA is not performed everywhere, and because indirect immunofluorescence is considered to be difficult to perform and operator‐dependent. For these reasons, the use of EmA has been largely replaced by the more easily available and reproducible assays for TTA. Interestingly, promising results have been shown for automated AI interpretation of EmA [44], which may in future help standardise and increase the availability of EmA testing. The excellent performance of EmA in our study reflects testing at a specialised centre with extensive experience, limiting the immediate generalisability of our findings to other settings. Our proposal for future study that coeliac disease could be diagnosed based on positive EmA in low‐risk adult patients might be supported by our data, but it also represents a significant departure from established guidelines [1, 2, 3, 4], so further study on more diverse multicentre cohorts will be necessary to ensure generalisability of our results. Centres without similar EmA expertise should carefully consider whether our results may apply to their clinical setting. Despite these limitations regarding EmA, we believe that our study provides initial valuable data on diagnostic outcomes based on patient clinical characteristics, which we believe generalise beyond the specific type of serological test that is employed, and can help inform the ongoing discussion on a no‐biopsy diagnostic strategy for adult coeliac disease. Regarding our choice of 45 years as an age cut‐off, this is also a limitation as it was based mainly on long‐term outcome data in coeliac disease [34] and expert opinion given the current lack of data in the literature on the relationship between clinical characteristics and diagnostic outcomes in patients with suspected coeliac disease, so future study is necessary to identify the best age cut‐off for biopsy‐sparing diagnosis of coeliac disease. Finally, although to avoid any potential selection bias we included in the study only patients directly diagnosed at our centre and excluded referred patients, we cannot fully exclude residual bias.

In conclusion, EmA has excellent diagnostic accuracy for coeliac disease in adults when performed at an expert centre and should be considered for future research on biopsy‐sparing diagnostic strategies for adult coeliac disease. Our findings support a biopsy‐sparing approach for diagnosing coeliac disease in selected adult patients with positive EmA, while emphasising the importance of continued endoscopic evaluation with duodenal biopsy in older patients with alarm symptoms to avoid missing non‐coeliac enteropathies. Based on our results, a biopsy‐sparing approach incorporating EmA could optimise resource utilisation and patient experience without compromising diagnostic accuracy. Future research is necessary to validate our findings on more diverse multicentre cohorts to ensure generalisability of our results before they can be incorporated into clinical practice.

## Author Contributions

**Stiliano Maimaris:** conceptualization, writing – original draft, methodology, writing – review and editing, formal analysis, data curation. **Annalisa Schiepatti:** conceptualization, writing – original draft, data curation, writing – review and editing. **Daniel Ignacio Conforme Torres:** data curation. **Roberta Muscia:** data curation. **Virginia Gregorio:** data curation. **Claudia Delogu:** data curation. **Ignazio Marzio Parisi:** data curation. **Michele Dota:** data curation. **Giovanni Arpa:** data curation. **Carolina Cicalini:** data curation. **Giulio Massetti:** data curation. **Chiara Scarcella:** data curation. **Paolo Minerba:** data curation. **Federico Biagi:** data curation, supervision, writing – review and editing, conceptualization.

## Conflicts of Interest

The authors declare no conflicts of interest.

## Authorship

*Guarantor of the article*: Prof. Federico Biagi.

## Data Availability

The data that support the findings of this study are available on request from the corresponding author. The data are not publicly available due to privacy or ethical restrictions.
